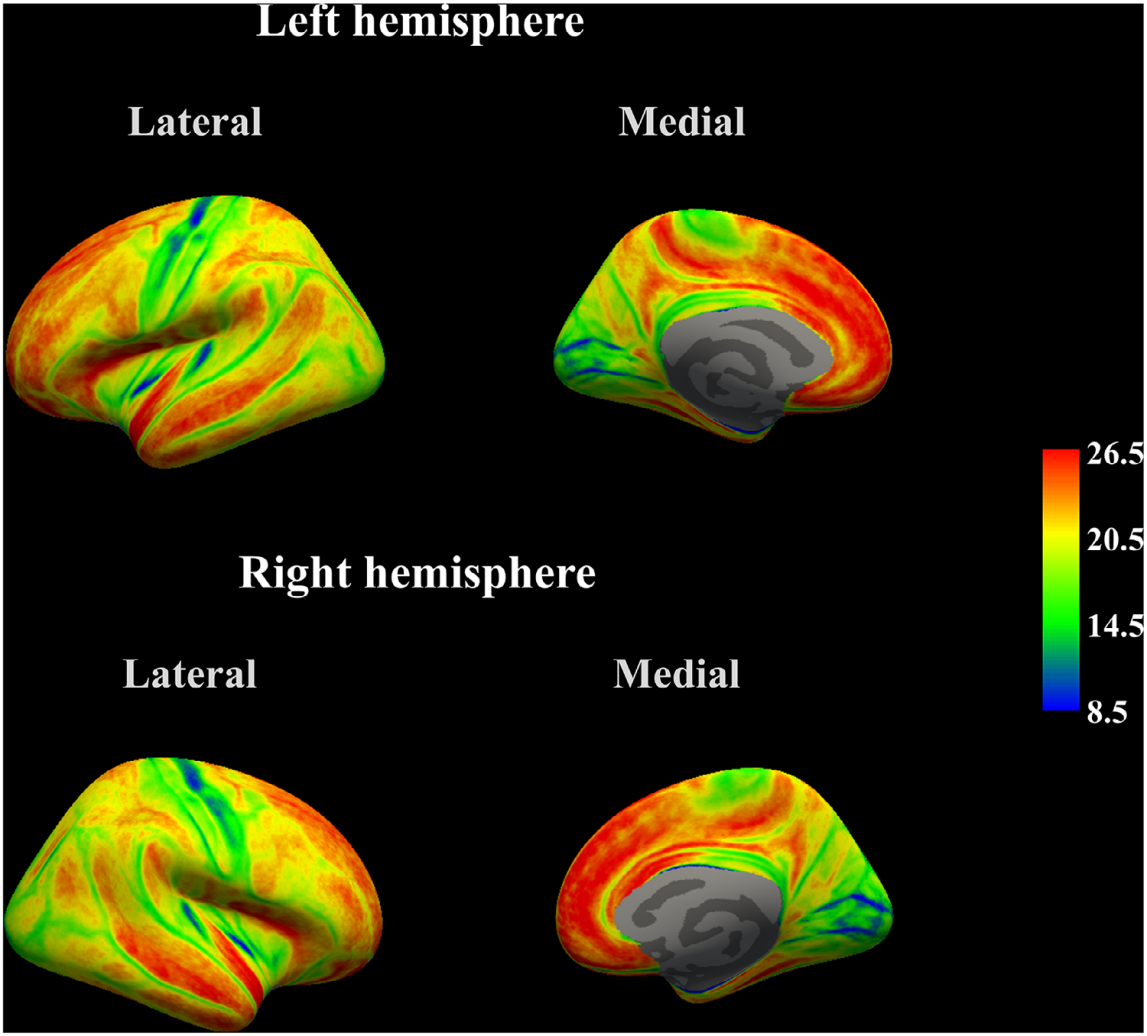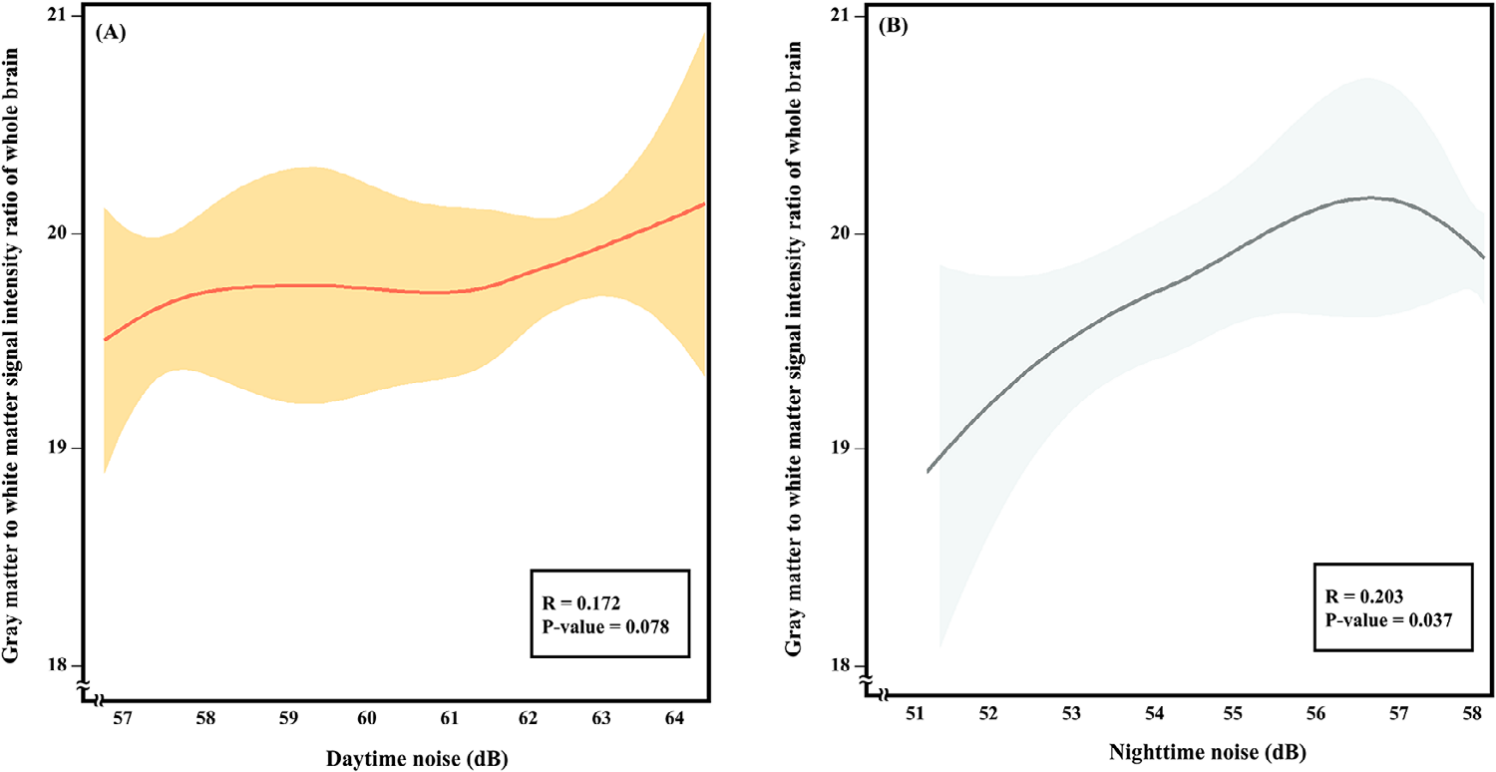# Environmental noise exposure and a new biomarker of neurodegeneration in Alzheimer's disease

**DOI:** 10.1002/alz70862_110001

**Published:** 2025-12-23

**Authors:** You Jin Kim, Seunghyun Lee, Jonn‐Yul Choi, Wanhyung Lee

**Affiliations:** ^1^ Chung‐Ang University, Dongjakgu, Seoul Korea, Republic of (South); ^2^ Pusan National University, Mulgeumup, Pusan Korea, Republic of (South); ^3^ Yonsei University, Wonju, Kangwon Korea, Republic of (South)

## Abstract

**Background:**

Environmental noise exposure is associated with neurodegenerative brain disorders. While observational studies have identified an association between environmental noise exposure and cognitive impairment, very few studies have examined the association by focusing on brain structure. This study aimed to demonstrate the relationship between environmental noise exposure levels and a new biomarker of neurodegeneration in Alzheimer's disease using the cortical gray to white matter signal intensity ratio (GWR).

**Method:**

After acquiring their 3D T1‐weighted images, 106 participants without cognitive impairment or neurological abnormalities were divided into the high‐ and low‐noise exposure groups, based on WHO‐recommended guidelines, and GWR values were calculated for each cerebral vertex using the T1‐weighted images. T‐test was conducted to identify significant differences between groups using whole‐brain and regional GWR values. The correlation between the GWR values in the regions that showed significant differences and noise exposure levels (daytime and nighttime noise) was analyzed based on the group comparison.

**Result:**

The whole‐brain GWR was significantly higher in the high noise exposure group (20.11 ± 0.93) compared to that in the low noise exposure group (19.68 ± 0.96, *p* = 0.036). The regional analysis showed that the left superior frontal gyrus, left caudal middle frontal gyrus, paracentral lobule, and precentral gyrus had significantly higher GWR values in the high‐noise exposure group than those in the low‐noise exposure group (*p* <0.05). Whole‐brain GWR values were significantly correlated with night noise levels (r=0.203, *p* = 0.037). Regional GWR values were positively correlated with both daytime and nighttime noise levels in regions that showed significantly higher values (*p* <0.05).

**Conclusion:**

Environmental noise exposure was significantly associated with GWR as a new biomarker of neurodegeneration in Alzheimer's disease. This study provides a useful explanation of the association between noise exposure and Alzheimer's disease.